# Connecting nutritional facts with the traditional ranking of ethnobotanically used fodder grasses by local farmers in Central Punjab of Pakistan

**DOI:** 10.1038/s41598-022-15937-6

**Published:** 2022-07-19

**Authors:** Nidaa Harun, Abdul Shakoor Chaudhry, Shabnum Shaheen, Mushtaq Ahmad, Zeynep Sahan, Hira Bashir

**Affiliations:** 1 Department of Botany, Faculty of Life Sciences, University of Okara, Okara, 56130 Pakistan; 2 School of Natural and Environmental Sciences, Newcastle University, Newcastle upon Tyne, NE1 7RU UK; 3 Department of Botany, Lahore College for Women University, Lahore, 54000 Pakistan; 4 Department of Plant Sciences, Quaid I Azam University, Islamabad, 45320 Pakistan; 5 Department of Veterinary, Kahta Vocational School, Adiyaman University, 02040 Adiyaman, Turkey

**Keywords:** Plant sciences, Ecology

## Abstract

The local farmers of Central Punjab, Pakistan have been using indigenous grasses as vital components of ruminant diets, but little is reported about their nutritional potential. Hence this study investigated nutritive potential of a selection of ethnobotanically important fodder grasses. Multiple nutritional parameters (proximate components, fibre fractions), secondary metabolites (phenolics, tannins) and in vitro digestibility values were determined. Furthermore, the legitimacy of ethnobotanical knowledge of local inhabitants about these grasses was also verified. The results suggested that majority (77%) of these grasses can be regarded as good quality fodders because of their high protein (169 g/kg) and good digestibility (457 g/kg) with moderate fibre (≤ 602 g/kg), lignin (≤ 50 g/kg) and secondary metabolites (total phenols ≤ 87 g/kg, total tannins ≤ 78 g/kg, condensed tannins ≤ 61 g/kg). Pearson correlation between nutritional parameters indicated that in vitro digestibility values were positively correlated with crude proteins (IVDMD, r = + 0.83 and IVOMD, r = + 0.83 respectively) and negatively correlated with fibre (NDF, r = − 0.91), ADF, r = − 0.84 and ADL, r = − 0.82) contents. Moreover, a positive relationship was identified between ethnobotanical knowledge and laboratory findings for studied grasses. Spearman correlation test showed that ranking of grasses based on ethnobotanical preferences were highly correlated (r values) with the laboratory results for CP (0.85), NDF (− 0.76), ADF (− 0.72) and ADL (− 0.62). The resilient complementarities between ethnobotanical preferences and nutritive analysis authenticate farmer’s traditional knowledge, which needed to be aligned with the corresponding scientific data. Farmers can use these findings for appropriate fodder selection and development of precise supplements for feeding ruminants within a sustainable and economically viable livestock industry for food security.

## Introduction

The Punjab province is the most developed, populous, and prosperous region of Pakistan, representing about 60% (110 Million) of the country's total population. It has an area of 205,344 sq km and it is the 2nd largest province in area after Balochistan. This zone is playing a promising role in agricultural production. It contributes about 68% to the annual food grain production, whereas 51 million acres of land is cultivated and another 9.05 million acres are remained to be converted as a cultivable land in different parts of this province (https://punjab.gov.pk/about_punjab_economy). Central Punjab refers to the alluvial planes that are surrounded by the two rivers i.e. Jhelum and Sutlej. This region is one of the biggest and most developed areas of Punjab, comprising one of the most extensive canal irrigation system frameworks in the world. This made it permissible for boosted agricultural output and an immense increment in arable land. Among agribusiness this land not only involves production of staple grains (wheat, maize, rice) but also plays a significant part to supply feeds ingredients for the livestock industry.

This region is one of great providers of ruminant milk and meat in the country. However indigenous farmers or shepherds of rural areas still rely on traditional fodders in order to raise their animals. Diversified range of plants such as trees, shrubs, herbs and grasses are traditionally significant for their fodder value^1^. Though all kind of floras are in use as fodders, the regional grasses are considered to be a more reliable fodder source for ruminant animals. This preference may be due to the fact that grasses are more palatable than other shrubby fodders for ruminants^1–5^. As grasses are able to grow massively in various seasons around the year, their accessibility for ruminant feeding is more convenient. It is reported that 53% of total ruminant feed is composed of grasses^6^. A recent ethnobotanical study of Central Punjab Pakistan had enlisted 53 valuable fodder grasses of this region^1^. This study also represented an order of priority among ethnobotanically listed fodder grasses which was based on their utilization preferences by local shepherds and animal caretakers.

Though Harun et al.^1^ highlighted many grasses as potential fodders based on their use by indigenous people to feed animals, this ethnobotanical data requires scientific verification. Thus, nutritional exploration of suitable forage species is strongly recommended for sustainable growth and reproduction of livestock^7,8^. Such kind of information will be supportive in planning to utilize these conventional fodders more appropriately to eliminate the nutritional inadequacies in animal feeding practices^9^. Around the globe the nutritional evaluation of ethnobotanical fodder and forages has been in practice^10–13^. In 2017, six ethnobotanically used grass species of western Maharashtra district Ahmednagar, India were nutritionally evaluated. It was reported that these grasses had relatively good levels of protein which could be a desirable contributor to an animal diet. However sometimes they are rich in silica which can negatively affect feed digestibility^14^. Another nutritional study conducted in 2004 in North Sumatra, Indonesia revealed that *Cynodon plectostachyus* was the most nutritious fodder among customarily used grass species^15^. Similarly in Bangladesh the nutritive value of three fodder grass species (*Brachiaria mutica*; *Echinochloa crusgalli* and *Hymenachne pseudointerrupta*) at different stages of maturity was evaluated. This study declared the studied grasses as a suitable fodder to feed ruminant animals^16^.

Nutritional evaluation of these ethnobotanically used fodders grasses is a global trend. However, in Pakistan only a few studies had been conducted in this regard and most of these studies were confined to Himalayan grassland^17,18^, highlands of Baluchistan^19^ and the desserts of Cholistan^20^. Moreover, with relevance to Central Punjab, Pakistan we found limited nutritional data about ethnobotanically used fodder grasses. Sultan et al.^21^ evaluated the nutritional capacitances of ten commonly used irrigated grasses (i.e. *Panicum antidotale*, *Sorghum halepense*, *Pennisetum purpureum*, *Vetiveria zizanioides Cymbopogon citrates*, *Cenchrus ciliaris Leptochloa fusca*, *Chloris gayana*, *Cynodon dactylon* and *Panicum colunum)*. Ahmed et al.^22^ also nutritionally investigated two traditionally used fodder grasses (*Eragrostis pilosa*, *Dactyloctenium aegyptium)* of district Sargodha. In another study five indigenous grasses (*C. ciliaris*, *L. fusca, C. gayana, C. dactylon and P. colonum*) of Faisalabad and Sargodha were subjected to proximate analysis and their order of preference was established on the basis of nutritional facts^23^. While these studies recommended many grasses as a valuable part of ruminant feed, they did not satisfactorily represent all the nutritional facts of a diversified range of ethnobotanically used fodder grasses in Central Punjab Pakistan.

Therefore, the current research was conducted as an extension of our previous studies^1^ to generate a nutritional database for the previously identified ethnobotanically used fodder grasses of Central Punjab Pakistan. We anticipate that this information and its subsequent use may help the herdsmen and range managers to determine the incorporation of suitable grasses in animal diets for an improved animal health and performance. Moreover, this study aimed to highlight possible complementarities between the fodder grass rankings based on ethnobotanical knowledge, preferences and nutritional compositions. The outcomes of this study could be used as a benchmark to exploit local fodders more efficiently as a nutritional resource to optimize animal production in an affordable and a sustainable manner.

## Results

### Nutritional composition

The overall nutritional compositions of studied grasses are presented in Table 1. A wide range of nutritional values were observed among selected grasses under this study. The moisture content of various grass species used for feeding livestock varied between 336 and 798 g/kg. Around 70% of the studied grasses contained more than 600 moisture g/kg. The maximum moisture content was observed in *Echinochloa crus-galli*, whereas minimum moisture was found in *Cenchrus setiger*. It is well-known that dry matter (DM) and moisture contents have inverse relationship to each other. Therefore, the grass species with peak moisture content had lowest DM i.e. 202 g/kg and species with minimum moisture possessed the maximum amount of DM i.e., 664 g/kg. Moreover, the ash and organic matter (OM) contents also have reverse relationship among them i.e., minimum ash with greater OM and vice versa. Hence current nutritional results reported the lower ash values in *Eragrostis minor* (7 g/kg) with upmost OM content (993 g/kg). Whereas *Setaria verticillata* exhibited the greater ash content (115 g/kg) with lowest OM content (885 g/kg). In regards to the fat content, *Acrachne racemosa* expressed the maximum value (46 g/kg) while *Agrostis gigantean* showed the least i.e., 20 g/kg. However, the crude protein (CP) contents in grasses ranged from 41 to 164 g/kg. The lower CP was observed in *Arundo donax* while higher CP was observed in *Eragrostis minor.* Interestingly these two species (*Arundo donax* and *Eragrostis minor*) exhibited exactly inverse results for NDF. *Eragrostis minor* reported least NDF (429 g/kg) whereas *Arundo donax* showed the highest value (798 g/kg). This showed a considerable negative association between CP and NDF contents of grasses. However, in terms of ADF, *Zea mays* showed the smallest value (247 g/kg) and maximum value was observed in *Setaria verticillata* (603 g/kg). The utmost ADL content was reported in *Arundo donax* (153 g/kg) although undermost ADL content was in *Brachiaria reptans* i.e. 35 g/kg.

**Table 1:** Nutritional contents, secondary metabolites and in vitro dry matter digestibility of all the studied ethnobotanical fodder/forage grasses.

Binomial name	ERG	Nutritional composition (g/kg)											Secondary metabolites (g/kg)			In vitro digestibility (g/kg)	
		M	DM	Ash	OM	Fat	CP	NDF	ADF	ADL	CE	HC	TP	TT	CT	IVDMD	IVOMD
*Agrostis gigantea* Roth	A	624	376	60	940	20	146	562	290	39	251	272	63	66	25	551	524
*Avena sativa* L	B	645	355	78	922	35	123	528	378	53	326	150	51	48	45	461	455
*Bromus japonicus* Thunb	A	633	367	68	932	30	140	548	333	33	300	214	45	36	21	529	534
*Dactylis glomerata* L	C	613	387	89	911	36	43	796	603	107	496	193	61	56	42	175	179
*Lolium temulentum* Linn	C	573	427	91	909	41	53	737	550	78	472	187	135	128	85	313	310
*Phalaris minor* Retz	A	675	325	65	935	35	129	527	364	36	328	162	50	37	31	484	479
*Poa annua* L	B	553	447	85	915	34	102	622	324	45	279	298	69	62	50	423	429
*Poa infirma* Kunth	B	693	307	86	914	33	95	638	373	45	328	264	64	53	42	424	429
*Polypogon monspeliensis* (L.) Desf	B	536	464	76	924	33	98	604	377	50	327	226	46	40	28	421	424
*Arundo donax* L	A	638	362	64	936	32	41	798	506	103	404	291	62	60	32	208	205
*Phragmites australis* (Cav.) Trin. ex Steud	C	713	287	74	926	42	116	608	448	50	398	160	128	119	52	455	448
*Aristida adscensionis* Linn	B	760	240	98	902	35	66	701	506	89	417	196	73	70	42	363	360
*Acrachne racemosa* (B. Heyne ex Roth) Ohwi	C	713	287	112	888	46	59	718	508	108	400	210	138	123	58	355	350
*Cynodon dactylon* (L.) Pers	A	601	399	65	935	29	161	444	298	34	264	146	49	38	29	639	645
*Dactyloctenium aegyptium* (L.) Wild	A	594	406	62	938	33	137	538	340	42	298	198	50	38	26	514	510
*Desmostachya bipinnata* (L.) Stapf	A	645	355	71	929	37	127	589	313	36	277	276	47	39	26	480	471
*Eleusine indica* (L.) Gaertn	A	645	355	63	937	38	159	482	262	37	224	220	40	28	20	623	632
*Enneapogon persicus* Boiss	B	540	460	69	931	34	47	730	554	100	454	177	46	42	36	334	330
*Eragrostis japonica* (Thunb.) Trin	B	579	421	58	942	34	99	652	376	49	327	276	68	55	44	440	441
*Eragrostis minor* Host	A	596	404	7	993	30	164	429	304	39	265	125	49	30	20	659	661
*Eragrostis pilosa* (L.) P. Beauv	B	619	381	66	934	33	108	511	306	48	257	205	63	59	41	443	448
*Leptochloa panicea* (Retz.) Ohwi	C	755	245	102	898	42	52	743	518	120	399	225	76	67	53	344	340
*Tetrapogon villosus* Desf	C	689	311	71	929	40	100	633	311	49	263	322	141	132	61	441	448
*Apluda mutica* L	C	675	325	80	920	41	63	718	522	72	450	196	153	129	71	360	352
*Bothriochloa bladhii* (Retz.) S.T. Blake	A	647	353	66	934	30	156	499	319	38	281	180	47	36	30	617	619
*Brachiaria ramosa* (L.) Stapf	B	613	387	71	929	31	98	631	488	41	448	143	69	64	46	467	471
*Brachiaria reptans* (L.) C.A.Gardner & C.E.Hubb	A	653	347	58	942	34	142	582	353	45	308	229	44	40	26	544	549
*Cenchrus biflorus* Roxb	B	677	323	68	932	23	104	606	338	43	294	269	67	59	47	430	435
*Cenchrus ciliaris* L	A	524	476	57	943	29	148	552	374	34	339	179	35	21	12	559	556
*Cenchrus pennisetiformis* Steud	A	624	376	58	942	26	137	568	274	42	231	294	38	26	17	519	510
*Cenchrus setiger* Vahl	C	337	663	63	937	44	98	603	423	52	371	180	122	109	72	421	428
*Chrysopogon aucheri* (Boiss.) Stapf	A	590	410	62	938	27	136	552	392	36	356	160	39	28	13	510	507
*Chrysopogon zizanioides* (L.) Roberty	A	579	421	56	944	22	135	599	326	39	287	273	37	29	19	502	509
*Cymbopogon jwarancusa* (Jones.) Schult	C	723	277	75	925	34	55	749	566	98	468	183	77	66	55	229	225
*Dichanthium annulatum* (Forssk.) Stapf	A	664	336	68	932	24	154	512	303	35	269	209	69	56	48	616	610
*Digitaria ciliaris* (Retz.) Koeler	B	659	341	61	939	33	97	632	393	47	347	238	70	65	58	428	432
*Digitaria longiflora* (Retz.) Pers	B	695	305	63	937	33	58	762	601	92	510	161	70	60	50	228	222
*Echinochloa colona* (L.) Link	B	746	254	60	940	38	103	618	371	41	330	246	78	72	59	426	424
*Echinochloa crus-galli* (L.) P. Beauv	B	798	202	60	940	28	106	560	299	48	252	260	63	59	45	435	439
*Heteropogon contortus* (L.) P Beauv. Ex. Roem & Schult	A	625	375	66	934	30	53	761	545	98	447	216	49	34	28	241	246
*Imperata cylindrica* (L.) Raeuschel	A	640	360	58	942	30	140	583	279	35	245	304	45	38	23	538	542
*Ochthochloa compressa* (Forssk.) Hilu	C	597	404	70	930	41	72	697	521	66	456	176	87	78	61	385	389
*Panicum antidotale* Retz	B	580	420	67	933	38	15	552	302	49	253	250	67	59	45	616	620
*Paspalidium distichum* L	B	546	454	66	934	36	109	531	313	38	275	218	70	61	49	449	441
*Pennisetum orientale* Rich	B	624	376	60	940	34	109	526	291	40	251	235	66	58	51	449	439
*Saccharum bengalense* Retz	A	613	387	56	944	30	132	522	310	34	276	213	52	50	37	499	491
*Saccharum spontaneum* L	A	656	344	53	947	24	126	512	241	40	201	271	62	47	34	471	474
*Setaria pumila* (Poir) Roem. & Schult	A	663	337	66	934	30	146	567	341	34	307	226	49	42	30	549	538
*Setaria verticillata* (L.) P. Beauv	C	661	339	115	885	40	53	744	502	73	429	242	139	132	65	226	223
*Setaria viridis* (L.) P. Beauv	A	515	485	59	941	36	149	532	245	40	205	287	41	29	12	610	602
*Sorghum bicolor* (L.) Moench	A	599	401	62	938	27	140	566	349	44	305	218	60	50	32	539	531
*Sorghum halepense* (L.) Pers	A	697	303	66	934	29	160	462	218	38	180	245	59	47	34	630	622
*Zea mays* L	A	634	366	63	937	29	164	452	229	34	195	223	68	70	33	641	635
mean		632	368	69	931	33	108	602	381	54	327	221	68	59	40	457	463

Moisture (M), dry matter (DM), organic matter (OM), crude proteins (CP), neutral detergent fibre (NDF), acid detergent fibre (ADF), acid lignin fibre (ADL), cellulose (CE), hemicellulose (HC), total phenolics (TP), total tannins (TT), condensed tannins (CT), In vitro Dry matter digestibility (IVDMD), In vitro organic matter digestibility (IVDMD), Ethnobotanical ranking groups (ERG) determined in the previous study of Harun et al.^1^, where A, B and C were identified as respectively high, moderate and low priority ethnobotanical grasses based on the experiences of local farmers.

### Secondary metabolites

In this current research, the anti-nutrient contents presented considerable variations in total phenols (TP), total tannins (TT) and condensed tannins (CT) i.e. 35–153 g/kg, 21–132 g/kg and 12–85 g/kg, respectively (Table 1). Among all the tested grass species, the maximum secondary metabolites were reported in *Cenchrus setiger*, *Phragmites australis*, *Acrachne racemosa*, *Lolium temulentum*, *Apluda mutica*, *Tetrapogon villosus* and *Setaria verticillata*. Conversely, the lowest values were recorded in *Cenchrus ciliaris* followed by *Cenchrus pennisetiformis, Chrysopogon aucheri, Eleusine indica Chrysopogon zizanioides*, *Setaria viridis* and *Eragrostis minor*.

### In vitro digestibility

Current digestibility analysis reported mean values of 457 g/kg and 463 g/kg for in vitro dry matter digestibility (IVDMD) and in vitro organic matter digestibility (IVOMD) respectively. The highest IVDMD and IVOMD were observed in *Eragrostis minor* (660 and 661 g/kg respectively) whereas the minimum values were observed in *Dactylis glomerata* (175 and 179 g/kg respectively) (Table 1).

## Discussions

As indicated in Table 1, the average moisture and DM contents were within the range for good quality fodders. However, it also depended upon the time, stage and weather of harvesting. The reported DM contents of *Cenchrus ciliaris*, *Cynodon dactylon*, *Apluda mutica*, *Setaria pumila*, *Saccharum spontaneum*, *Desmostachya bipinnata* and *Pennisetum orientale* were found to be much higher in comparison to the values presented by Sultan et al.^24^ who analyzed some grasses from Northern regions of Pakistan. The probable reasons for this variation are disparities in the land geography, climate, soil composition and sampling times which can affect the corresponding nutritional variations in forages^25–28^. However, in comparison to the current study, Manzoor et al.^23^ and Sultan et al.^21^ reported lower DM values for some grasses (*Cenchrus ciliaris*, *Cynodon dactylon, Panicum antidotale*, *Sorghum helepense* and *Chrysopogon zizanioides*) from the same geographical range. These lower values were possibly due to differences in soil fertility level, phenological states of grasses, and sampling or analytical procedures.

It is worthwhile to know the amount of total ash in different feeds because it contains different types and amounts of macro, micro and trace minerals which are needed for ruminant animal’s health^29^. This information can help us to use suitable forages for feeding animals to satisfy their mineral requirements with or without supplements. Conversely, high mineral contents in animal feeds may dilute the quantity of other available nutrients to animals. Moreover, if animal fed with excessively high mineral contents, these will not only affect the digestibility but also might cause the accumulation and crystallization which ultimately lead to urinary restrictions or infections. In the current study mean ash and OM contents were found to be within the satisfactory limits (Table 1). Sultan et al.^24^ and Rafay et al.^20^ stated much higher ash values for *Cenchrus ciliaris*, *Cynodon dactylon*, *Setaria pumila*, *Saccharum spontaneum*, *Pennisetum orientale Cymbopogon jwarancusa*, *Ottochloa compressa* and *Panicum antidotale.* Variation in soil and other habitat features might have affected the ash content of plants^30^. However, the current ash values were also found to be lower than the reported values from similar agro climatic zone^21,23^. These lower ash values inferred the probable less soil contamination of these samples^31^.

The current study reported that the average fat content was 33 g/kg which is within acceptable limits for ruminant diets (Table 1). This amount reasonably satisfies the ruminant’s fat requirement. In fact, high concentrations of dietary fats can not only suppress the rumen microbial (protozoa and fungi) activity but also it can reduce the fibre digestion and DM intake in ruminant animals^32^.

This study found that out of 53 studied fodder grass, 40 species possessed fairly moderate to good CP range i.e. 169–95 g/kg. Generally, the upper limit of CP values in present analysis were found to be relatively higher than the maximum CP content of some previously studied grass species from similar zone^21,23^. These disparities in CP content were probably due the difference in their growth stages and soil fertility at the time of sampling^23,33^. However comparable CP values were reported by other researchers^31,34–36^, who obtained variable CP contents (up to 170 g/kg) of several tropical grasses around the globe. The ruminant’s protein demand varies according to its age or growth stage, such as the higher CP levels (150–160 g/kg) are especially good for lactating cows because during lactation amino acids demand is higher for additional metabolic functions and synthesis of milk protein^37^. Current results revealed that *Dichanthium annulatum*, *Bothriochloa bladhii*, *Eleusine indica*, *Sorghum helepense*, *Cynodon dactylon*, *Zea mays* and *Eragrostis minor* were especially good in this aspect because of their higher CP content. However, for non-lactating ruminants comparatively lower protein intake is recommended. Therefore, measurement of CP is a supportive tool in order to assure that animal is ingesting sufficient proteins as per its body requirement. As intake of excessive proteins or consumption of poor quality proteins are both economically detrimental^29^.

The recommended critical value for CP content is 70 g/kg^38^. Results showed that CP of *Ottochloa compressa* (72 g/kg) was at borderline; whereas remaining 12 grasses possessed CP lower than the critical value (Fig. 2). They showed decreasing CP contents from 60 to 40 g/kg and their descending order was arranged as *Aristida adscensionis* > *Apluda mutica* > *Acrachne racemosa*** > ***Digitaria longiflora* > *Cymbopogon jwarancusa* > *Lolium temulentum* > *Setaria verticillata* > *Heteropogon contortus* > *Leptochloa panacea* > *Enneapogon persicus* > *Dactylis glomerata*** > ***Arundo donax.* By comparing these results with ethnobotanical priority groups of fodder grasses (previously reported by Harun et al.^1^ it was shown that two (*A. donax* and *H. contortus*) of those grasses belonged to the high priority group (A) and three (*A. adscensionis, E. persicus, D. longiflora*) were from moderate priority group (B) whereas rest of the seven grasses were from the low priority grass group (C). The presence of greater number of fodder grasses with lower CP in group C declares a supportive evidence for ethnobotanical knowledge of local inhabitants of the study area. The intake of this kind of protein deficient diet will not only result in the loss of appetite but also reduce the cellulolytic activity of rumen microbes which slow down the fibre digestion^39^. Low feed intake and poor digestion reduce the food efficiency which can cause underprivileged growth and development of livestock^40,41^. Therefore, for healthy animal productivity uninterrupted supply of CP is mandatory^42,43^. Although 77% of studied grasses showed sufficient levels of CP which recommend them as good protein sources for maintaining healthy livestock, they still cannot compete with high CP containing legumes^44,45^. The differences between grass and legumes for CP contents are quite significant; hence a blend of grass and legume in ruminant diet is more recommendable^34,46^.

**Figure 1:**
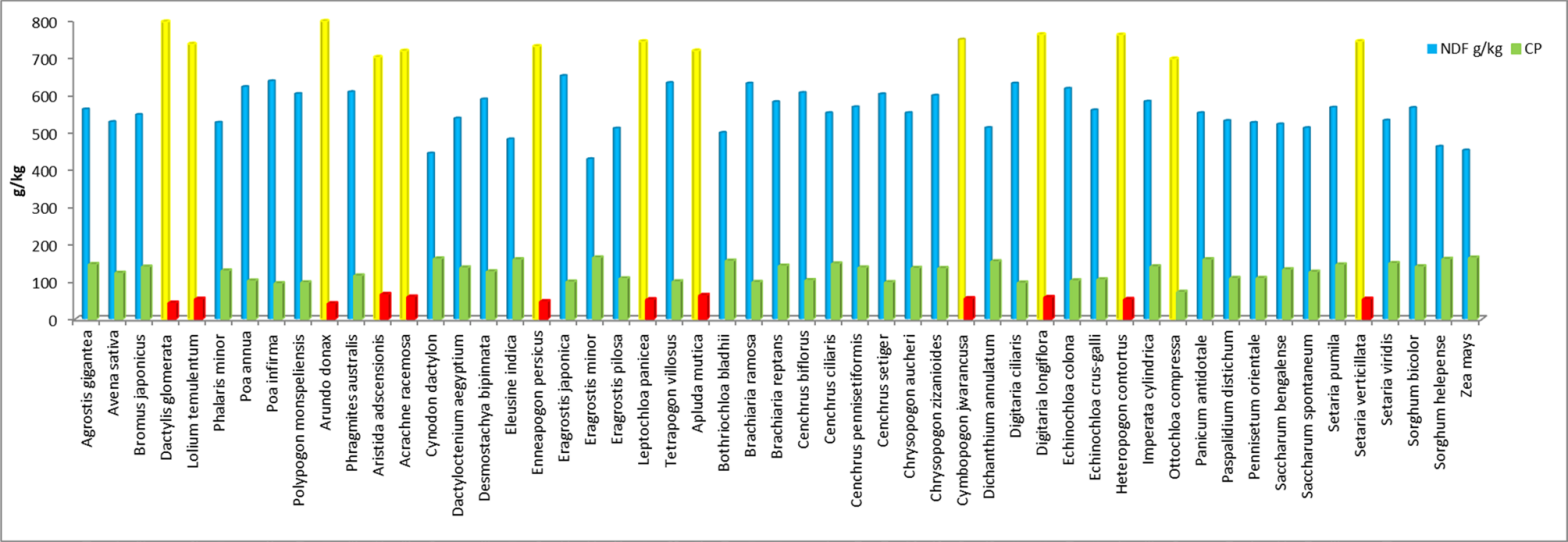
Comparative illustration of the CP and NDF contents of ethnobotanical grasses (Yellow NDF bars showing above critical value > 650 g/kg while red CP bars showing below critical value < 70 g/kg).

Fibre is often regarded as the negative index of nutritive quality^47,48^. It is negatively related to digestibility of feed, as lower the concentration of fibre in feed it become easier to digest which results in good energy value^24^. Fibre can be determined in terms of NDF and ADF contents but the NDF method is considered to be most reliable one for measuring the total cell wall a fibre contents in a feed^29^. Forage digestibility and ruminant’s consumption capacity largely influenced by its NDF content^49^. Its value in animal feed is quite critical and a feed is considered to be of poor quality if it has NDF above 650 g/kg^50^. The current study revealed the mean NDF value of 602 g/kg which is under the critical limit but 12 species i.e., *Arundo donax* > *Dactylis glomerata* > *Digitaria longiflora* > *Heteropogon contortus* > *Cymbopogon jwarancusa* > *Setaria verticillata* > *Leptochloa panacea* > *Lolium temulentum*** > ***Enneapogon persicus* > *Apluda mutica* **> ***Acrachne racemosa* > *Apluda mutica* > *Aristida adscensionis* had NDF above the critical value (650 g/kg). However, *Ottochloa compressa* exhibited the NDF values that were very close to the critical value of 697 g/kg. The grasses with more than the critical NDF value indicated reduction in voluntary feed intake and feed conversion efficiency but lengthier rumination periods^51^. Norton^37^ stated that NDF varying from 670 g/kg to 780/kg was considered to be high enough to limit DM intake and digestibility. The results predicted an interesting relationship between CP and fibre contents as indicated in Fig. 1. This figure showed grasses with higher CP were lower in NDF.

NDF is also associated with other cell wall structural contents i.e., lignin, cellulose and hemicellulose^52^. Lignin content is also negatively correlated with fodder palatability^42,53^ because it has negative affect on OM digestibility^54^. The average ADL content was reported as 382 g/kg. One of the significant reasons in preference of grasses by ruminants is their digestible cellulose and hemicellulose contents. The microflora of ruminant’s digestive is capable to efficiently digest cellulose and hemicellulose of grasses^42^. The mean cellulose and hemicellulose in studied grasses were reported as 317 g/kg and 220 g/kg, respectively. However, digestibility of cellulose is inversely proportional to the amount of lignin. As fodder matures its lignification increases with 100 g/kg which lessens the cellulose digestibility by about 60%. The lignin content in younger fodder is the 50 g/kg, which increases the cellulosic digestibility up to 80%^55^. The current results exhibited that 75% of species had lignin from 34 to 50 g/kg which represented their good cellulosic digestibility. However a few species with higher lignin content (*Brachiaria ramosa*, *Phragmites australis*, *Ottochloa compressa*, *Apluda mutica*, *Dactylis glomerata*, *Lolium temulentum*, *Leptochloa panicea*, *Digitaria longiflora*, *Heteropogon contortus*, *Cymbopogon jwarancusa*, *Enneapogon persicus*, *Arundo donax*, *Setaria verticillata*. *Acrachne racemosa*, *Aristida adscensionis*) negatively affected the cellulose digestion in ruminants. On comparison with previous studies relatively higher fibre values (NDF, ADF, ADL, cellulose and hemicellulose) were found in the findings stated by Rafay et al.^20^ and Sultan et al.^24^. The soil fertility and season of fodder harvesting are important influencing factors for variation in fibre contents^56–58^.

**Figure 2:**
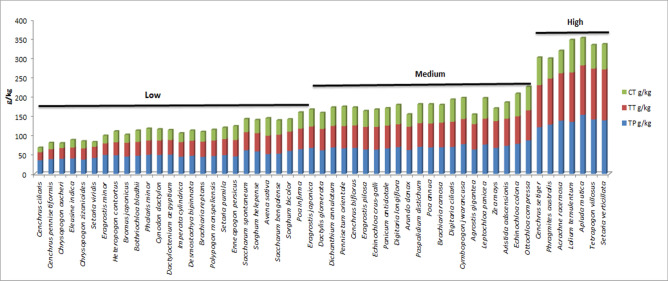
Clustering of ethnobotanical fodder grasses based on their secondary metabolite contents i.e. high (> 100 g/kg), moderate (50–100 g/kg) and low (< 50 g/kg). Here, greater number of species were in low (24) followed by moderate (22) and high (7) grass clusters.

Secondary metabolites like phenolics and tannins are known as anti-nutritional factors because of their negative effects on ruminant health especially if consumed in large amounts^59–62^. The significant possible damages of secondary metabolites are such as reduction in immune function, growth and reproduction impairments, which ultimately leads to animal morbidity and mortality^63,64^. By considering these effects, quantification of secondary metabolites is appraised to be essential in order to make appropriate fodder selection^65^. According to reports, tannins at 60–120 g/kg DM in animal diet can lower the efficiency of animal digestive system and ultimately its productivity as well^60^. However, few other researchers reported that low levels of these secondary metabolites positively affected ruminant health^66,67^. Like condensed tannins between 20 and 50 g/kg DM are essentially required for efficient utilization of nitrogen and healthy weight maintenance^66^. On the basis of recommended acceptable ranges of secondary metabolites, the studied grass species had been categorized into three groups i.e. high (> 100 g/kg), moderate (50–100 g/kg) and low (< 50 g/kg). Figure 2 shows that greater number of species were in low (24) followed by moderate group (22) and high (7). Grass species ranked as low and moderate groups are within the satisfactory limits for animal health. These results suggested that 86% of studied grass species possessed fewer secondary metabolites than their suggested toxic levels which indicated that those were potentially of good quality as animal fodders. Certain levels of phenols in these species are beneficial as antioxidative, anti-inflammatory, anti-diabetic, anti-allergy, anti-microbial and gastro or hepato-protective activities^68^. Moreover, sufficient tannin concentrations facilitate to increase the digestion proficiency, milk production, wool growth and animal’s ovulation rate^69^. Additionally, specific quantity of these secondary metabolites has positive ecological effects such as improvement of soil quality and nutrient cycles^69^. However, grasses belong to the high secondary metabolite category (*Cenchrus setiger*, *Phragmites australis Acrachne racemosa*, *Lolium temulentum*, *Apluda mutica*, *Tetrapogon villosus* and *Setaria verticillata*) are not considered good for ruminant’s health. Panhwar^64^ stated that the excessive intake of secondary metabolites (anti nutrients) could affect the digestibility of vital nutrients which not only lessened the palatability but could also be fatal. It had been reported that higher dosage of phenols may reduce the bone mineralization and could also cause disturbance in cholesterol or estrogen levels^70–72^. Whereas greater amounts of tannins could result in reduction of protein digestion and absorption which eventually depress the voluntary feed intake (VFI)^73,74^.

**Figure 3:**
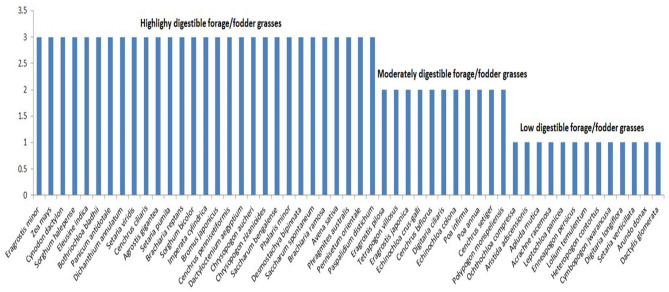
Clustering of ethnobotanical fodder/grasses based on their in vitro digestibility i.e. high (< 450 g/kg), medium (400–450 g/kg) and low digestible fodder grasses (> 400 g/kg). Results showing greater number of grass species (n = 29) in high digestible cluster followed by low (n = 13) and medium (n = 11) digestibility clusters.

As reported above, numerous nutritional parameters can indicate the potential feed values for providing particular nutrients. However, the real feed value for animal can only be determined by finding the digestion of those nutrients. However, the digestibility of fodder or forages were strongly affected by the seasonal variations^75^. Kallah et al.^76^ and Megersa et al.^77^ suggested that on average 450 g/kg digestibility of forage/fodder was sufficient to maintain an optimal animal performance. The findings of Van Soest^78^ suggested that 400–700 g/kg digestibility for grasses were found in tropical and subtropical lands. Noguiera^39^ also acclaimed 400–527 g/kg digestibility for grasses. For more understanding, ethnobotanical grasses were further classified into 3 clusters i.e. high (< 450 g/kg), medium (400–450 g/kg) and low digestible grasses (> 400 g/kg) (Fig. 3). Results reported greater number of grass species (n = 29) in high digestible cluster followed by low (n = 13) and medium (n = 11) digestibility groups. Minor in vitro DM digestibility indicates the presence of anti-nutritional factors within those fodders which can obstruct the activity of microbial activities in rumen^24,79,80^.

### Correlation between studied nutritional parameters

Evitayani et al*.*^15^ also predicted similar negative relationship between CP and fibre contents while studying nutritive value of tropical forages in North Sumatra, Indonesia. This clearly indicates that if any species possessed high protein content then its NDF, ADF, ADL and cellulosic contents must be lower. Especially the negative connection between CP and NDF contents was also supported by Ronquillo et al*.*^81^ and Mlay et al*.*^82^. Whereas with the exception of hemicellulose, all other studied fibre contents (NDF, ADF, ADL and cellulose) exhibited significant positive relationship with each other.

Many factors influenced the digestibility of a particular fodder. Harper and McNeill^49^ described that forage digestibility was negatively influenced by NDF contents whereas Arif et al.^83^ argued about the positive influence of CP on digestibility values of fodder/forages. As mentioned in Table 2, IVDMD and IVOMD values were positively correlated with CP (r = + 0.83 and r = + 0.83 respectively) and negatively correlated with NDF (r = − 0.91), ADF (r = − 0.84) and ADL (r = − 0.82 in studied fodders grasses. These results can be interpreted as that by increasing the crude protein the digestibility amplified, however increase in cell wall content lessened the digestibility of a particular fodder. These correlation findings were in line with the previous findings^21,24,84,85^. These results were also concordant with the report of Van Soest^47^ who stated negative association between NDF and digestibility of various feeds. Lichtenberg and Hemken^86^ specified that DM digestibility was decreased by 3–4 units with per unit increase in lignin content. However, poor digestibility fodder grasses can be improved by adding urea supplements into an animal diet^65^. Some other studies also supported the fact that by addition of urea as a supplement can improve the digestibility of low quality forages^87,88^.

**Table 2:** Pearson correlations (r) (alongside their significance levels at *, **, ***) between crude proteins (CP) and overall fibre content neutral detergent fibre (NDF), acid detergent fibre (ADF), acid lignin fibre (ADL), cellulose (CEL), hemicellulose (HCL), total phenol (TP) total tannins (TT), condensed tannins (CT), in vitro dry matter digestibility (IVDMD), in vitro organic matter digestibility (IVOMD) (g/kg) of studied ethnobotanical fodder**/**forage grasses (n = 53).

Items	CP	NDF	ADF	ADL	CE	HC	TP	TT	CT	IVDMD	IVOMD
CP		− 0.856***	− 0.786***	− 0.819***	− 0.735***	0.009	− 0.477***	− 0.495***	− 0.607***	0.832***	0.827***
NDF			0.893***	0.859***	0.855***	0.045	0.442**	0.458**	0.488***	− 0.914***	− 0.914***
ADF				0.849***	0.989***	− 0.409**	0.419**	0.418**	0.489***	− 0.844***	− 0.844***
ADL					0.761***	− 0.144	0.361**	0.368**	0.405**	− 0.818***	− 0.820***
CE						− 0.461**	0.413**	0.410**	0.486***	− 0.806***	− 0.805***
HC							− 0.035	− 0.001	− 0.096	0.020	0.021
TP								0.987***	0.845***	− 0.422**	− 0.424**
TT									0.857***	− 0.442**	− 0.447**
CT										− 0.528***	− 0.526***
IVDMD											0.999***

Here *, ** and *** represent significance at P < 0.05, P < 0.01 and P < 0.001 respectively.

Moreover, it also had been observed that digestibility was inversely proportional to secondary metabolite contents (anti-nutritional factors). As shown in Table 2. The IVDMD and IVOMD values showed negative association with TP (r = − 0.422 and − 0.424 respectively), TT (r = − 0.442 and − 0.447 respectively) and CT (r = − 0.528 and − 0.526 respectively). Njidda^89^ supported this fact that low level of tannins resulted in good IVDMD of fodders. This is because those tannins in NDF and ADF firmly bound to cell wall and cell protein and thus resulted in lower digestibility values^51^. Whereas CT had also been notorious in order to lower the digestibility values^54^.

### Correlation between ethnobotanical knowledge and laboratory findings

**Figure 4:**
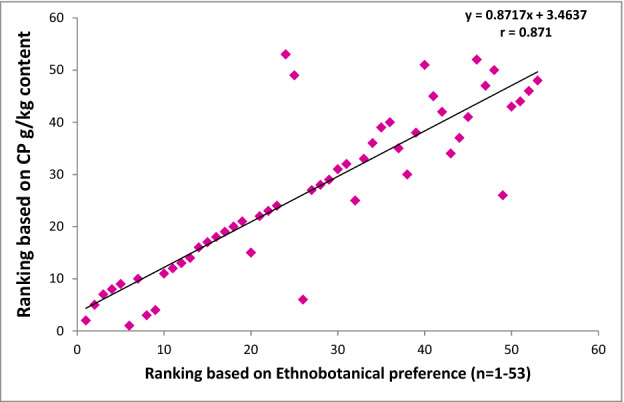
Positive correlation (r) between ethnobotanical preferences ranking of fodder grass versus order of priority based on CP content (high to low).

**Figure 5:**
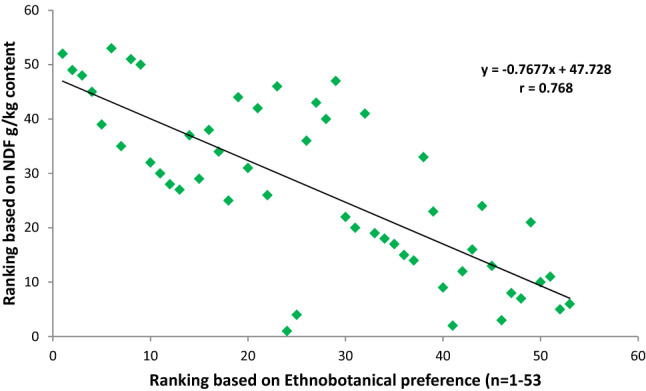
Negative correlation between fodder grass rankings based on ethnobotanical preference ranking versus ranks based on NDF contents (high to low).

**Figure 6:**
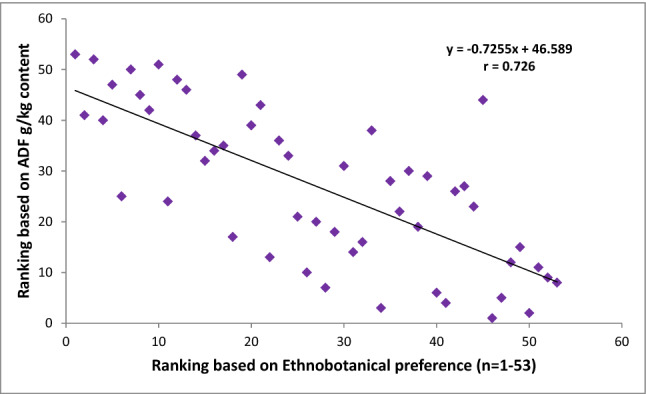
Negative correlation between fodder grass rankings based on ethnobotanical preference ranking versus ranks based on ADF contents (high to low).

**Figure 7:**
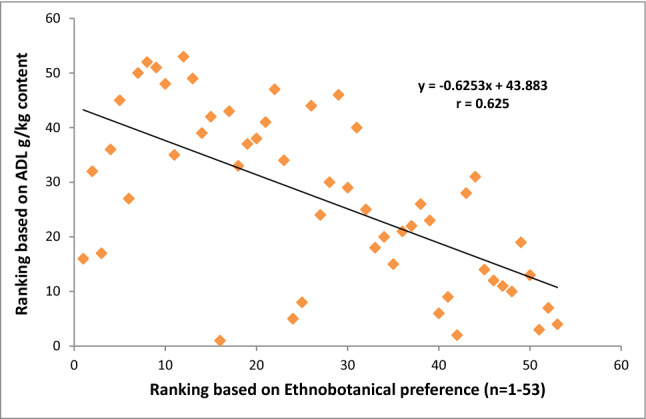
Negative correlation between fodder grass rankings based on ethnobotanical preference versus ranks based on ADL contents (high to low).

**Figure 8:**
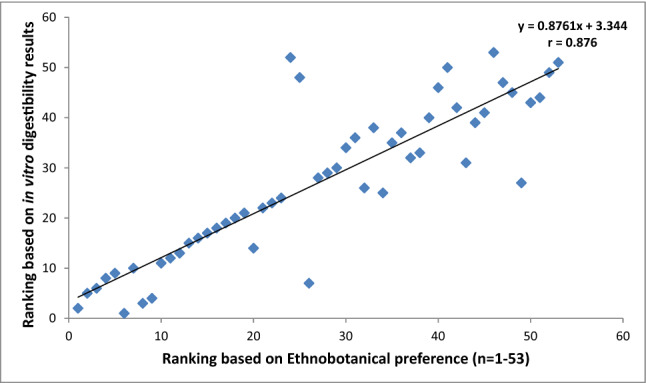
Positive correlation between fodder grass rankings based on ethnobotanical preference versus order of priority based on in vitro digestibility (high to low).

After obtaining the nutritional results, all the studied grasses were ranked in order of priority with reference to CP, NDF, ADF, ADL, IVDMD and IVOMD contents. These nutritional rankings were compared with the ethnobotanical preferences ranking of fodder/forage grasses established earlier in the previous study of Harun et al.^1^ (Figs. 4, 5, 6, 7, 8). The results demonstrated strong correlations between laboratory findings and ethnobotanical knowledge about studied forages/fodder grasses, perceived by local respondents. Figure 4 showed a strong positive association (r = + 0.8) between ranking based on CP and ethnobotanical preferences. This means that grasses which ranked as a high priority by local communities also possessed sufficient quantities of proteins and fodder grasses with low priority were below the critical CP value (> 70 g/kg). However, as CP and fibre contents were inversely proportional to each other, ranking order based on NDF, ADF and ADL exhibited negative correlation (r = − 0.768, r = − 0.726, r = − 0.625 respectively) with the ethnobotanical preferences of fodder grasses (Figs. 5, 6, 7). This can be inferred that a fodder/forage grass with high fibre content is least preferable by the local farming communities of Central Punjab Pakistan. Also, the ranking based on the digestibility results of fodder grasses positively correlated (r = + 0.876) with the ethnobotanical rankings of these grasses (based on the experiences of local people) (Fig. 8).

Results of crosstab analysis reported strikingly positive associations between the clusters of grasses based on laboratory results (in vitro digestibility and secondary metabolites) and ethnobotanical ranking groups of high, medium and low priority. Secondary metabolites analysis also showed an affirmative association with ethnobotanical knowledge. Crosstab analyses clearly declared that grasses ranked in high and medium ethnobotanical priority groups were comparable to low and medium secondary metabolites ranking groups (Table 3). Additionally, those grasses which were least preferred ethnobotanically lie in the category of high secondary metabolites (anti-nutrients) group. This can be explained as the grasses of high and medium ethnobotanical fodder priority possessed secondary metabolites (total phenols, total tannins and condensed tannins) below the critical limit whereas grasses with least ethnobotanical fodder priority contained comparatively higher amount of secondary metabolites. Moreover, Crosstab analysis of in vitro digestibility and ethnobotanical ranking groups of grasses showed that out of 25 high priority ethnobotanical grasses, 23 grasses were also positioned in high priority group based on the in vitro digestibility values. Similarly, out of 11 low priority ethnobotanical grasses group, 8 grasses were also placed in low priority group of in vitro digestibility (Table 4). The study revealed that the ethnobotanical knowledge of local shepherds and animal caretakers was quite consistent with the nutritional data of studied grasses. The current findings are in agreement with the reported data of Keba et al.^10^, Dhungana et al.^11^, Talore^12^, Rakib-Uz-Zaman et al.^90^ and Rodrigues et al.^91^ who supported the positive association between laboratory results and ethnobotanical knowledge. Therefore, traditional knowledge should not be ignored and must be used as an approach for better understanding of nutritive potential of local fodder/forage resources under predominant environmental conditions. However, the laboratory based nutrient compositions are needed to formulate nutritious diets to optimize the use of local feed resources to promote livestock health and the environment.

**Table 3:** Crosstab analyses between secondary metabolites based grass categories and ethnobotanical ranking groups of studied ethnobotanical fodder grasses.

Ethnobotanical ranking groups	Secondary metabolites based grasses categories			Total
	High	Medium	Low	
High (A)	0	4	21	25
Medium (B)	0	14	3	17
Low (C)	7	4	0	11
Total	7	22	24	53

**Table 4:** Crosstab analyses between In vitro digestibility’s based grasses categories and ethnobotanical ranking groups of studied ethnobotanical fodder grasses.

Ethnobotanical ranking groups	In vitro digestibility based grasses categories			Total
	High	Medium	Low	
High (A)	23	0	2	25
Medium (B)	5	9	3	17
Low (C)	1	2	8	11
Total	29	11	13	53

## Conclusions

Grasses of Central Punjab Pakistan had been used as animal fodder for centuries but unfortunately little information about their nutritional worth was available. This study not only provided a nutritional profile of studied grasses but also made an attempt to validate the ethnobotanical knowledge of local inhabitants of Central Punjab Pakistan about these fodder grasses. It can be concluded that a strong positive correlation existed between ethnobotanical preferences of fodder grasses and nutritional facts of particular species. Nutritional results confirmed that those grasses which ranked superior by the local inhabitants of the study area also contained higher CP levels than those species which were ranked as inferior. Moreover, the grasses perceived for low palatability possessed great levels of structural fibres (NDF, ADF and ADL) and secondary metabolites (anti-nutrients). The Pearson correlation studies also showed that grasses with higher proteins had low fibre content and good digestibility. It can be summarized that good quality fodders were high in protein and digestible nutrients, but low in fibre and lignin. The strong complementarities between ethnobotanical preferences and nutritive analysis reflected the reliability of ethnobotanical knowledge of local farmers and shepherds. However, it is recommended to integrate these conventionally used fodders into modern feeding systems. The good quality grasses can be directly incorporated into ruminant diets whereas grasses with low nutritional quality can be improved by using biochemical processing before making either silage or hay or can be mixed with either good quality forages or supplements before feeding the animals. These results are valuable in making appropriate fodder selection and supplement development that will match livestock requirements which consequently can support economical livestock performance. However, further studies are required to evaluate their mineral composition; feed intake and animal’s ability to efficiently utilize these ethnobotanical feed resources for sustainable animal production in low to moderate animal input systems.

## Methods

### Study area and its main features

**Figure 9:**
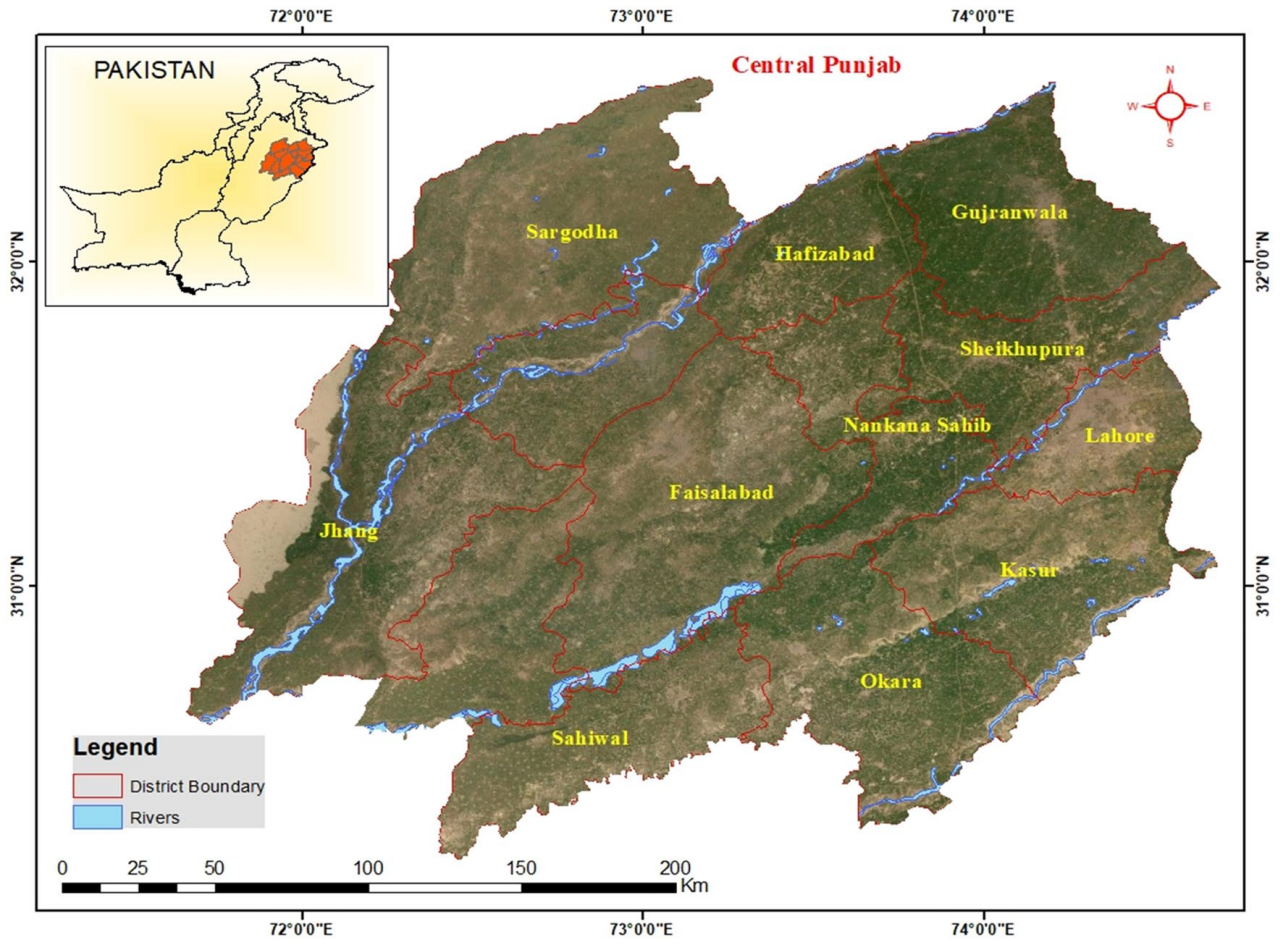
Illustration of sample collection sites i.e. Sargodha, Sialkot, Lahore, Gujranwala, Faisalabad, Okara, Sahiwal, Jhang, Sheikhupura, Nankanasab rcGIS version 10.8 software was to draw this map. Basemap is added by choosing the online basemap option in ArcGIS. https://www.esri.com/en-us/arcgis/products/arcgis-pro/overview.

The study was conducted in Central Punjab region of Pakistan which is regarded as the subtropical continental low land (Fig. 9). Normally temperature of this region remains hot but also shows significant variation between summers and winters. On average, the summer temperature ranged between − 2° and 45 °C whereas in winters it can drop down to − 10 °C. The mean annual rainfall of this area is 46 cm. This region consists of 19 cities which are categorized under 3 agro-ecological zones i.e., Northern irrigated zone, Sandy deserts zone and Barani zone. For the current study Northern irrigated zone was selected because the dominant grasses in this zone were a result of irrigation by using an extensive canal system and a good precipitation. Most of the time ad libitum grazing was in practice but cut and carry system for mixing specific forages with other feed types was also used in these regions^1^.

### Sampling of fodder grasses

For sample collection the rural areas from Northern irrigated zone were actually targeted because of their reliance around feeding conventional grasses for raising their ruminant animals. As the literature reported, the grasses were more palatable before their maturity. Therefore, representative samples of each of the 53 specimens from different parts of the same field were collected at their pre bloom stage through repeated field visits during this study. These samples represented the same fodder grasses as previously reported by Harun et al.^1^. Representatives of collected fodder grass sample was taxonomically identified by using specimen identification guidelines in the herbarium of Lahore College for Women University, Lahore and the Quaid i Azam University, Islamabad. Moreover two online plant databases i.e. flora of Pakistan (http://www.efloras.org/index.aspx) and flora of India (https://sites.google.com/website/efloraofindia/) and various grass flora identification keys^92^ were also used in fodder grass identification process. However, remaining samples were washed to remove contaminants or dust particles, shade dried and finely ground (Ultracentrifugal mill ZM 200). All the samples were kept inside airtight polythene bags until further nutritional analysis as described below.

### Nutritional analysis

Parameters like moisture, dry matter, ash, proteins, fat, were evaluated by following the standard methods of AOAC^93^. Nitrogen content was determined by the advance MACRO CUBE System and obtained nitrogen value was multiplied by 6.25 to estimate the crude protein (CP) contents of these samples. However, for fat analysis advance micro-digester (MARS6) was used. Whereas neutral detergent fibre (NDF), acid detergent fibre (ADF), acid lignin fibre (ADL) were done by adopting the methodology prescribed by Van Soest et al*.*^48^. Cellulose and hemicellulose contents were calculated by the formulae Cellulose = ADF-ADL, Hemicellulose = NDF-ADF^48^.

### Secondary metabolites analysis

The selected secondary metabolites i.e. total phenols (TP), total tannins (TT) and condensed tannins (CT) that can affect the fodder quality were also estimated by using the methodology prescribed by Makkar et al.^94^. For the estimation of TP, TT and CT tannic acid and epigallocatechin gallate was used respectively to establish relevant standard curves.

### In vitro digestibility analysis

The method of Tilley and Terry^95^ was adopted for digestibility test by using only the first stage of the 2 stage procedure. Rumen fluid (RF) was collected from 3 freshly slaughtered cattle (*Aberdeen Angus* breed) at a local abattoir (Linden Foods, Ltd.) of Newcastle upon Tyne, UK. Equal volumes of these RF were pooled and then mixed with the pre warmed buffer solution in a ratio of 1:2 and kept at 39 °C in a water bath (Gallenkamp UK Ltd) in order to maintain anaerobic conditions. Moreover, carbon dioxide was flushed into this buffered RF (BRF) and pH was adjusted around 7. For the incubation 0.5 g of each ground fodder grass sample was put inside the 50 ml capacity polypropylene tubes and 40 ml of the BRF was also dispensed into each tube. These tubes were sealed with the rubber stoppers (equipped with gas pressure discharge valves) and incubated in a water bath at 39 °C. The tubes were shaken manually few seconds thrice a day (morning, afternoon, evening). After 48 h the tubes were taken out and placed inside an ice filled bucket to stop the on-going fermentation. Later on the tubes were subjected to centrifugation (accuSpinTM3R) at 2000 rpm for 10 min. The insoluble residues were washed, dried, weighed and then ashed before estimating In vitro dry matter digestibility (IVDMD) and In vitro organic matter digestibility (IVOMD). IVDMD of samples were measured by drying the washed residues at 80 °C whereas IVOMD was estimated by formation of ash in the furnace at 550 °C. The calculations were done by following the formulae mentioned below and a blank sample of BRF was used for correction in dry matter residue weight.

$$\text{IVDMD}= (\text{sample weight}-\text{ corrected residue DM weight}) \times 1000$$

$$\text{IVOMD}= [(\text{sample DM}-\text{ corrected non}-\text{degradable OM weight}) /\text{ sample DM}] \times 1000$$

### Ranking of studied fodder grasses

After obtaining laboratory results (nutritional, secondary metabolites and digestibility) the fodder grasses (n = 53) were ranked in descending order (high to low) according to results of each studied parameter. For each parameter, grass at rank 1 considered as most potential one whereas at rank 53 with the lowest potential. This ranking helped to correlate the laboratory findings with ethnobotanical preferences.

### Statistical analysis

The obtained data was computed in the excel sheets and graphical illustrations were made for data analysis. Correlation between CP, NDF, ADF, ADL, CE and HC were inferred by using Pearson correlation method (r < 1; P < 0.05) through SPSS version 23. Moreover, the Spearman’s rank correlation (r < 1; P < 0.05) was also used to examine possible relationships between laboratory results and ethnobotanical preferences of fodder and grazing grasses at P < 0.01. Crosstab method within descriptive statistics of SPSS was also employed for making comparisons between secondary metabolites based grass categories and ethnobotanical ranking of studied fodder grasses. Additionally, comparisons were made between In vitro digestibility based grass categories and ethnobotanical ranking of studied fodder grasses. Microsoft Excel was used to organize different data sets for creating trend lines and visual presentations.

## Data Availability

The datasets used and/or analyzed during the current study are available from the corresponding author on a reasonable request.
